# Electrochemical metallization cell with anion supplying active electrode

**DOI:** 10.1038/s41598-018-30746-6

**Published:** 2018-08-22

**Authors:** Ziyang Zhang, Yaoyuan Wang, Yan Luo, Yuhan He, Mingyuan Ma, Rongrong Yang, Huanglong Li

**Affiliations:** 1 Department of Precision Instrument, Center for Brain Inspired Computing Research, Tsinghua University, Tsinghua, China; 2 Department of Electronic Engineering, Tsinghua University, Tsinghua, China

## Abstract

Electrochemical metallization (ECM) memories are among the various emerging non-volatile memory technologies, contending to replace DRAM and Flash and enabling novel neuromorphic computing applications. Typically, the operation of ECM cell is based on the electrochemical redox reactions of the cation supplying active electrode (e.g., Ag, Cu). Although extensively investigated, the possibility of utilizing new materials for the active electrode remains largely undiscussed. In this paper, an ECM cell with a Te active electrode is fabricated. It is found that the SET operation of the device occurs under negative voltage on the active electrode, which is opposite to that of the device with Ag electrode, indicating that the Te electrode supplies Te^2−^ anions by electrochemical reduction. The influence of the electrolyte material on the switching properties is also found to be more significant for devices with Te electrodes. For Pt/GeS/Te and Pt/Ge_2_Sb_2_Te_5_/Te cells, repeatable unipolar and bipolar resistive switching are observed, respectively, which can be attributed to the rupture of the filament by Joule heating for the former and by ECM for the latter in the RESET process. The semiconducting properties of Te, the reversed operating polarity and the electrolyte dependent switching characteristics open up unprecedented prospects for ECM cells.

## Introduction

To address the demands imposed by big data and mobile electronics, non-volatile memory (NVM) technologies emerge and lead the data storage paradigm shift^1–5^. The material systems that have been investigated for the NVMs have also received considerable attention from the neuromorphic research community, aiming to enable new computing modalities inspired by the biological brains^6–10^. Among various NVM technologies, ECM memories hold the advantages of easy fabrication, fast operation, low power consumption and the scalability down to atomic level^2,11^. ECM cell relies on the redox reactions of the active electrode (AE) and ion movement in the electrolyte, which leads to the reversible resistive switching between the high resistance state (HRS) and low resistance state (LRS) by the formation (from HRS to LRS, SET operation) or rupture (from LRS to HRS, RESET operation) of the nanoscale conductive filament(s) inside the electrolyte layer^11^. The ECM cells can naturally mimic many features of the biological synapses since the latter have similar operation principle, namely, the release of chemical particles into the junctions^6^. The typical ECM cell has a cation supplying active electrode (c-AE), Ag or Cu, which undergoes electrochemical oxidation during the SET operation and supplies Ag^+^ or Cu^2+^ cations^11^. Cations then move toward the inert counter electrode (CE), and are subsequently reduced (metallization) and nucleate, forming metallic filament(s) within the electrolyte. The ECM process is field dependent, so the applied voltage must be positive on the c-AE in order to set the device^11^. On the other hand, the RESET operation requires negative voltage on the c-AE to rupture the filament(s), resulting in bipolar resistive switching.

A broad spectrum of materials have been considered for the ECM cells and their influences on the resistive switching characteristics have been studied. Yang *et al*.^12^ reported that ion mobilities and redox rates, which are highly electrolyte and ion species dependent, govern the filament growth modes and structures. Valov *et al*.^13^ reported that the chemistry and transport properties of the materials system determine the nanobattery effect in ECM cells, which has strong impact on the dynamic behavior of the devices. Tappertzhofen *et al*.^14^ reported that the catalytic activity of the CE towards the water redox process determines the concentration of dissolved ions within the electrolyte. The nanoionic kinetics has also been compared between devices with Ag and Cu c-AEs^15^. The overwhelming majority of these works, however, focused on materials for the electrolyte and CE but discussed little on the possibility of utilizing new materials other than Ag and Cu for the AE. A limited number of works have demonstrated that alternative metals such as Ta and Ti can serve as the c-AE^16^. With the goal of overcoming the limitation on usable AE materials, Song *et al*.^17^ have reported ECM cell modality based on symmetric inert electrodes structure with sandwiched Ag-doped electrolyte.

Recently, Yoo *et al*.^18^ reported the bipolar switching in Pt/Ge_2_Sb_2_Te_5_/Pt cell which is attributed to the formation and rupture of Te semiconducting filament by the ECM mechanism. In analogy to the ECM cells, unintentionally introduced Te layer near one of the inert Pt layers serves as the AE. Unlike the conventional ECM cells with c-AEs, Te electrode supplies Te^2−^ anions by electrochemical reduction since the SET operation of the device occurs under negative voltage on the AE, which is opposite to that of the device with Ag electrode. In addition, Te is semiconducting and thus the cell kinetics shows different temperature dependence than that of the conventional ECM cells in which metallic filaments are formed. The semiconducting properties of Te and its one-dimensional Van der Waals structure have also enabled novel transistor devices^19^. Moreover, Te has other appealing properties, such as photoconductivity^20^, thermoelectricity^21^ and piezoelectricity^22^, for applications in sensors, optoelectronics and energy devices. Therefore, introducing Te as the anion supplying active electrode (a-AE) into the ECM cell not only offers new modality of the ECM memory technology but also opens up unprecedented applications based on the integrated multi-functionalities of Te. In this paper, we fabricate Pt/GeS/Te and Pt/Ge_2_Sb_2_Te_5_/Te cells. The resistive switching characteristics are compared with the corresponding device counterparts with Ag c-AEs and the electrolyte dependence is investigated.

## Results and Discussion

**Figure 1:**
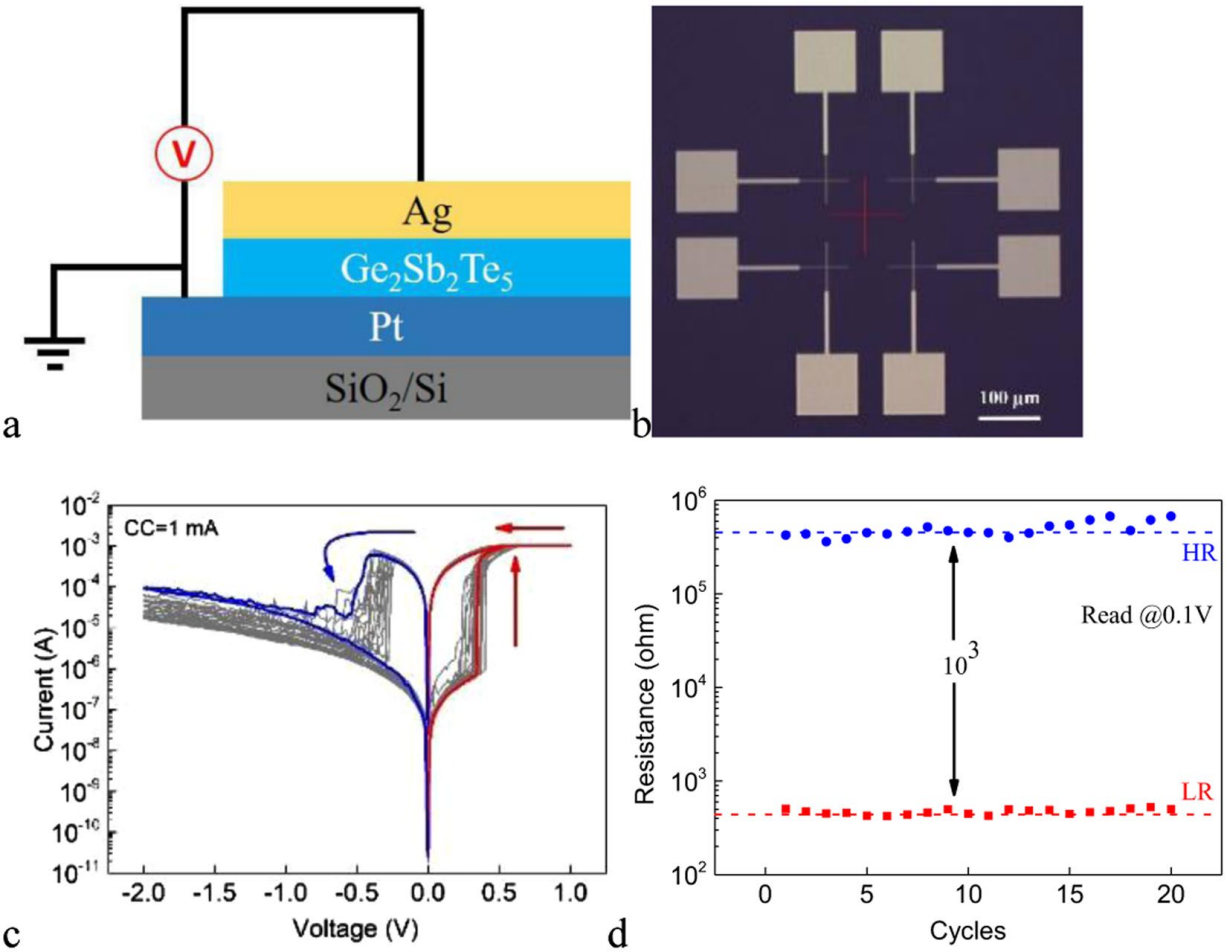
(**a**) The schematic structure of the Ag/Ge_2_Sb_2_Te_5_/Pt cell. (**b**) Optical image of the device with 2 × 2 μm^2^ junction area. (**c**) The DC I-V curves of 20 consecutive sweep cycles for the Ag/Ge_2_Sb_2_Te_5_/Pt cell under the SET CC of 1 mA. The positive direction in the diagrams of this work corresponds to positive voltage on the top electrode. (**d**) The LR and HR of the Ag/Ge_2_Sb_2_Te_5_/Pt cell for 20 consecutive sweep cycles. The voltage of read pulse is 100 mV.

We first fabricate the typical Ag/Ge_2_Sb_2_Te_5_/Pt cell where Ag is used as the c-AE. Figure 1(a) shows the schematic structure of the cell. The optical image of the device with 2 × 2 μm^2^ junction area is shown in Fig. 1(b). The DC I-V curves of 20 consecutive sweep cycles under the SET compliance current (CC) of 1 mA for cell with 2 × 2 μm^2^ junction area are shown in Fig. 1(c). The pristine cell is in the HRS. The voltage on the c-AE is first swept in the positive direction. When the voltage reaches ~ + 0.3 V, the cell switches to the LRS abruptly. Reversing the polarity of the voltage, the cell switches back to the HRS at ~ −0.4 V in a gradual manner. The voltage required to SET the pristine device is similar to those in the subsequent cycles. In other words, the device is forming free. Figure 1(d) shows the low resistance (LR) and high resistance (HR) for these 20 sweeps. It can be seen that the cell shows stable switching endurance and the resistance ratio between the HRS and the LRS is larger than 10^3^. The observed bipolar resistive switching originates from the electrochemical redox reactions of the c-AE and the cation movement in the electrolyte, leading to the formation or rupture of Ag metallic filament(s) within the electrolyte.

**Figure 2:**
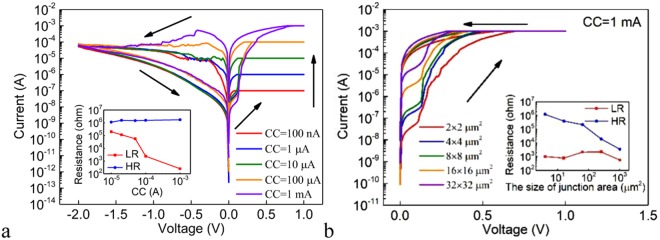
(**a**) The DC I-V curves under the different SET CCs for the Ag/Ge_2_Sb_2_Te_5_/Pt cell. Inset: the dependence of the LR and HR on the CC for the Ag/Ge_2_Sb_2_Te_5_/Pt cell. (**b**) The DC I-V curves of the SET processes for Ag/Ge_2_Sb_2_Te_5_/Pt devices with different sizes of the junction areas under the SET CC of 1 mA. Inset: the dependence of the LR and HR on the size of the junction area for the Ag/Ge_2_Sb_2_Te_5_/Pt cell.

The DC I-V characteristics for the Ag/Ge_2_Sb_2_Te_5_/Pt cell under different SET CCs are shown in Fig. 2(a) and the inset shows the dependence of the LR and HR on the CC. The LR decreases with increasing CC, implying the formation of stronger conductive filament(s) under higher CC. The relationship between the HR and the end voltage during the negative sweep, or the negative stop voltage (NSV) is also investigated (Fig. S1(a,b)). Under small NSV, there is no RESET phenomenon and the device remains in the LRS. Under large enough NSV that allows the RESET to occur, the HR of the device increases exponentially with increasing NSV. The monotonic dependence of the HR on the NSV can be expected from the gradual nature of the RESET switching, as can be seen from Figs 1(c) and 2(a). Figure 2(b) shows the DC I-V characteristics in the SET process for devices with different sizes of the junction areas under the SET CC of 1 mA. It can be seen that the cell switch to the LRS at ~ + 0.3 V regardless of the sizes of the junction areas. The inset of Fig. 2(b) shows the dependence of the LR and HR on the size of the junction area. The HR decreases with increasing size of the junction area, whereas the LR remains almost constant due to the filamentary conduction. Therefore, further reduction of the size of the junction area is expected to improve the on/off ratio.

**Figure 3:**
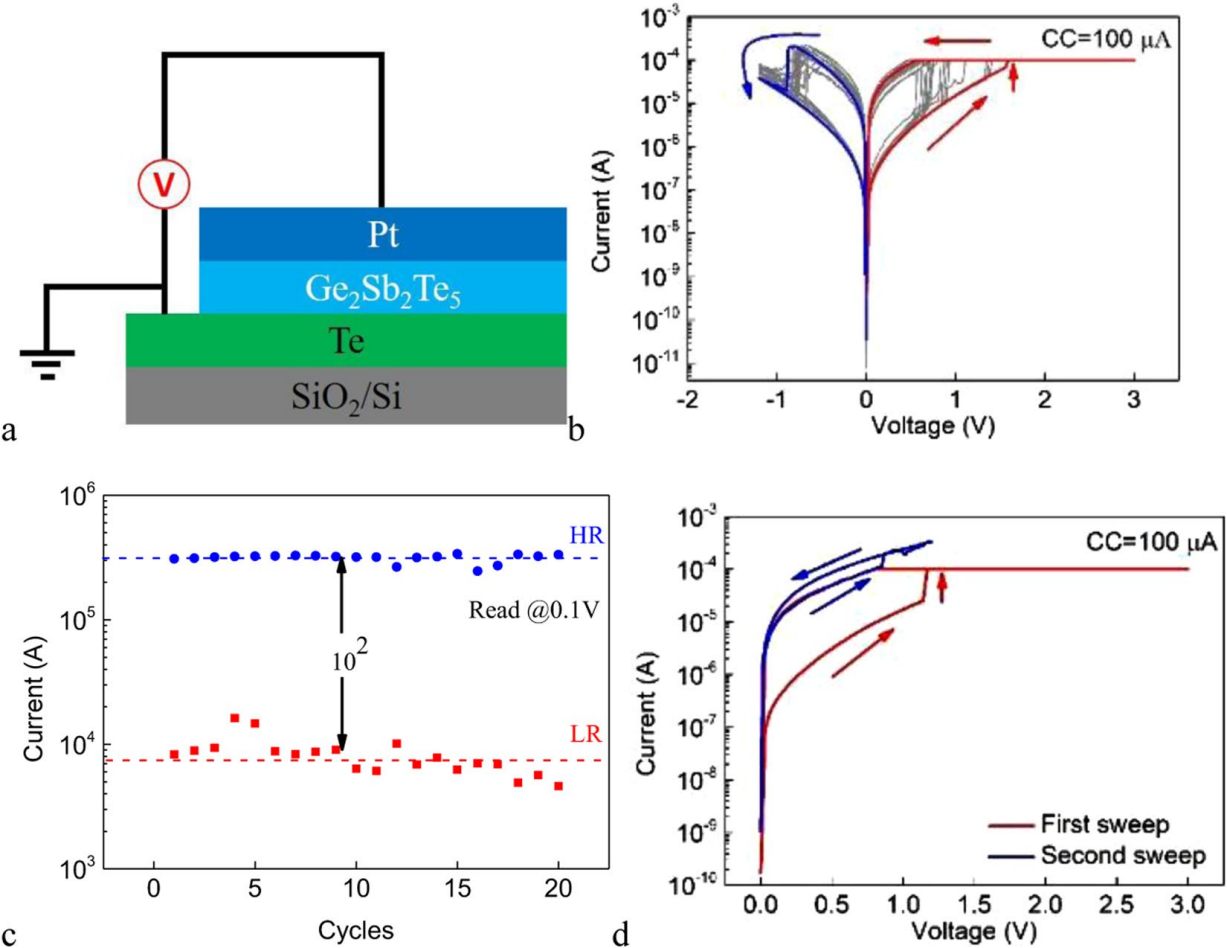
(**a**) The schematic structure of the Pt/Ge_2_Sb_2_Te_5_/Te cell. (**b**) The DC I-V curves of 20 consecutive sweep cycles for the Pt/Ge_2_Sb_2_Te_5_/Te cell under the SET CC of 100 μA. (**c**) The LR and HR of the Pt/Ge_2_Sb_2_Te_5_/Te cell for 20 consecutive sweep cycles. The voltage of read pulse is 100 mV. (**d**) The DC I-V curves of two consecutive voltage sweeps only in the SET direction. The SET CC is 100 μA.

To study the impact of a-AEs on the resistive switching characteristics, we fabricate the Pt/Ge_2_Sb_2_Te_5_/Te cell. Figure 3(a) shows the schematic structure of the cell. Figure 3(b) depicts the DC I-V curves of 20 consecutive sweep cycles under the SET CC of 100 μA for cell with 2 × 2 μm^2^ junction area. The cell also shows repeatable bipolar resistive switching behavior. However, the voltage polarities relative to the AE for the SET and RESET processes are opposite to those of the Ag/Ge_2_Sb_2_Te_5_/Pt cell, respectively, namely, SET (RESET) occurs under the negative (positive) voltage on the a-AE. In addition, much larger voltage is required to SET the pristine devices, compared with the SET voltages in the subsequent cycles. In other words, the Pt/Ge_2_Sb_2_Te_5_/Te device requires electroforming program, in contrast to the Ag/Ge_2_Sb_2_Te_5_/Pt device. We attribute the difference between these two kinds of devices to the different ionic radii of Ag^+^ and Te^2−^. The radius of Ag^+^ (1.15 Å) is much smaller than that of Te^2−^ (2.21 Å), which leads to the much easier diffusion of Ag into the electrolyte. It is also noted that the absolute value of the set voltage is larger than that of the Ag/Ge_2_Sb_2_Te_5_/Pt cell. The difference of the set voltages between the Ag/Ge_2_Sb_2_Te_5_/Pt and Pt/Ge_2_Sb_2_Te_5_/Te cell is expected due to the unique chemical natures of semiconducting Te. It has been reported that different electric fields are required for the ECM processes of different metals due to the different electrochemical dynamics^12^, which is also electrolyte dependent^23^. Figure 3(c) shows the LR and HR for 20 consecutive sweeps. The cell shows stable switching endurance and the resistance ratio between the HRS and the LRS is larger than 10^2^. The lower resistance ratio than that of the Ag/Ge_2_Sb_2_Te_5_/Pt cell may be rationalized by the higher LR of the Pt/Ge_2_Sb_2_Te_5_/Te cell as expected due to the semiconducting rather than metallic property of the Te filament.

Ciocchini *et al*.^24^ have reported that native ion migration in Ge_2_Sb_2_Te_5_ is also possible to induce bipolar resistive switching. To verify that the observed switching in our devices is due to the presence of the Te anion supplying electrode, a control Pt/Ge_2_Sb_2_Te_5_/Pt device is fabricated and its switching behavior is studied (see Fig. S2). The results indicate that it is not the Ge_2_Sb_2_Te_5_ electrolyte but the Te electrode that is responsible for the switching.

To confirm the voltage polarity dependent switching behavior, we apply consecutive voltage sweeps only in the SET direction under the SET CC of 100 μA. As is shown in Fig. 3(d), no RESET switching occurs. It is also found that no resistive switching occurs if positive voltage on the a-AE is applied to the pristine device (not shown). These indicate that the SET and RESET processes require exclusive voltage polarities. In addition, Fig. 3b shows that the peak currents of the SET and RESET processes are of the same order of magnitude. These facts indicate that the SET and RESET processes are predominantly electric field driven or electrochemical: when negative voltage is applied on the Te a-AE, Te is electrochemically reduced to Te^2−^ anions which migrate toward the Pt CE under the electric filed, and are subsequently oxidized back to Te and nucleate, leading to the formation of Te semiconducting filament(s) and the decrease of cell resistance. When changing the voltage polarity, the reversed redox reactions occur, leading to the dissolution of Te filament(s) and the restoration of the HR of the cell. It should be pointed out that the thermal effects are not excluded during the switching processes. In particular, conducting paths exist during the initial stage of the RESET process and therefore the local temperature could increase significantly due to the Joule heating effect which facilitates the disconnection of the filament by the diffusion of Te anions into the surroundings under their concentration gradient. The switching mechanisms are schematically shown in Fig. S3.

**Figure 4:**
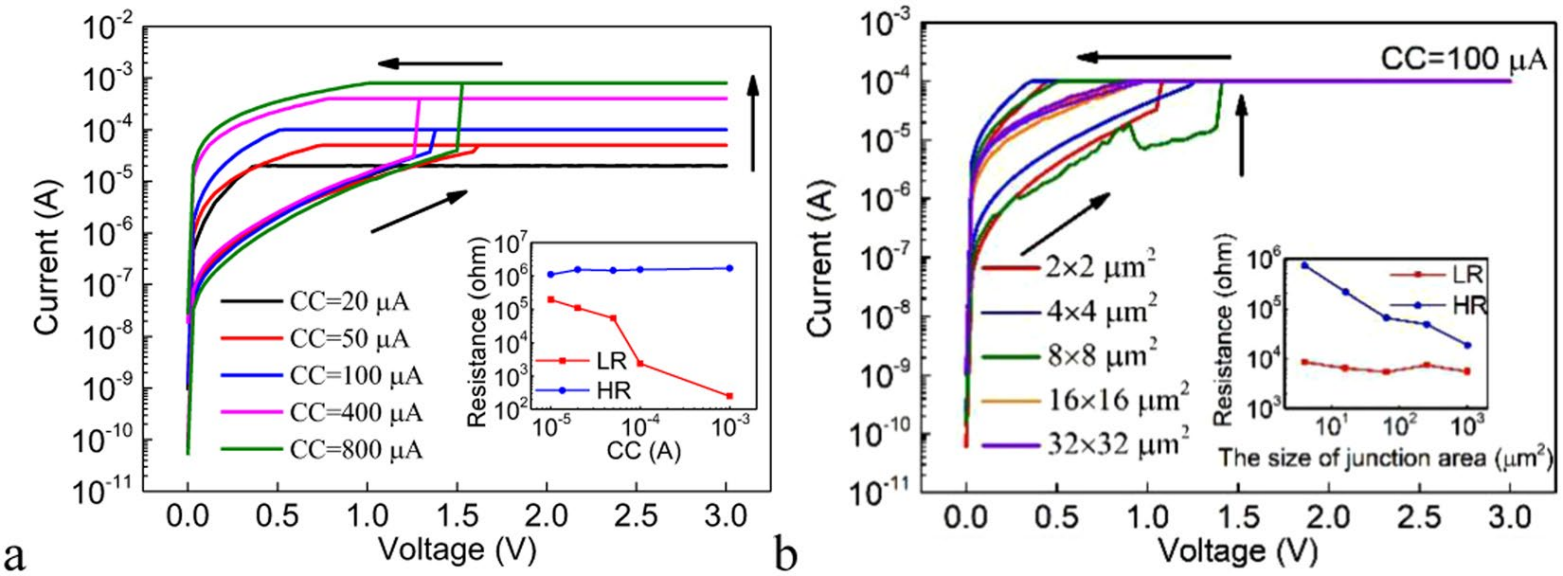
(**a**) The DC I-V curves of the SET processes under different CCs for the Pt/Ge_2_Sb_2_Te_5_/Te cell. Inset: the dependence of the LR and HR on the CC for the Pt/Ge_2_Sb_2_Te_5_/Te cell. (**b**) The DC I-V curves of the SET processes for Pt/Ge_2_Sb_2_Te_5_/Te devices with different sizes of the junction areas under the SET CC of 100 μA. Inset: the dependence of the LR and HR on the size of the junction area for the Pt/Ge_2_Sb_2_Te_5_/Te cell.

Figure 4(a) shows the DC I-V characteristics for the Pt/Ge_2_Sb_2_Te_5_/Te cell under different SET CCs and the inset shows the dependence of the LR and HR on the CC. The LR decreases with increasing CC due to the formation of stronger filament(s). The relationship between the HR and the NSV is also investigated (Fig. S1(c,d)). Under small NSV, there is no RESET phenomenon and the device remains in the LRS. Under large enough NSV that allows the RESET to occur, the resistance values are of the same order of magnitude, irrespective of the NSV. This is unlike the above shown Ag/Ge_2_Sb_2_Te_5_/Pt device. The independence of the HR on the NSV can be expected from the abrupt nature of the RESET switching, as can be seen from Figs 3(b) and 4(a). Figure 4(b) shows the DC I-V characteristics in the SET process for devices with different sizes of the junction areas under the SET CC of 100 μA. The cells switch to the LRS between 1.2 V and 1.6 V. The inset of Fig. 4(b) shows the dependence of the LR and HR on the size of the junction area. The LR is independent of the size of the junction area, remaining almost constant due to the filamentary conduction, whereas the HR increases with decreasing size of the junction area.

For a selected AE material, the ECM cell kinetics has also been found to be strongly influenced by the electrolyte^12,23^, resulting in a plethora of resistive switching behaviors. To investigate the electrolyte dependent switching characteristics of the devices with c-AE and a-AE, GeS electrolyte is used for comparison.

To avoid misinterpretation of the origin of the resistive switching of the GeS based cells, we first fabricate the Pt/GeS/Pt cell and find no resistive switching under both voltage polarities up to 3 V absolute voltage value (not shown). This rules out the possibility of native ions induced resistive switching.

**Figure 5:**
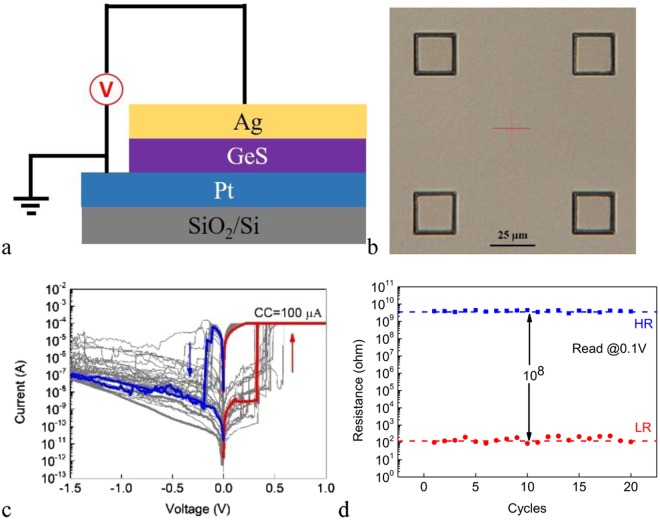
(**a**) The schematic structure of the Ag/GeS/Pt cell. (**b**) Optical image of the device with 24 × 24 μm^2^ junction area. (**c**) The DC I-V curves of 20 consecutive sweep cycles for the Ag/GeS/Pt cell under the SET CC of 100 μA. (**d**) The LR and HR of the Ag/GeS/Pt cell for 20 consecutive sweep cycles. The voltage of read pulse is 100 mV.

Figure 5(a) shows the schematic structure of the Ag/GeS/Pt cell. The optical image of the device with 24 × 24 μm^2^ junction area is shown in Fig. 5(b). We use larger sizes of the junction areas for the GeS based devices than those for the Ge_2_Sb_2_Te_5_ based ones to increase the success rate of the fabrication. As will be seen, the filamentary nature of the switching or the electrode size independent LR has also been verified for the GeS based devices. Therefore, the use of larger electrode sizes will not affect the conclusion.

Figure 5(c) shows the DC I-V curves of 20 consecutive sweep cycles under the SET CC of 100 μA for cell with 24 × 24 μm^2^ junction area. The voltage on the Ag c-AE is first swept in the positive direction. The cell switches to the LRS abruptly at ~ + 0.4 V. The subsequent negative sweep switches the cell back to the HRS at certain voltage in the range between −0.2 V and −0.4 V. The device is forming free. It is also found that the set voltage is larger than that of the Ag/Ge_2_Sb_2_Te_5_/Pt cell, which may be attributed to the different cation mobilities and redox rates in Ge_2_Sb_2_Te_5_ and GeS electrolyte materials^23^. The much higher HR of the Ag/GeS/Pt device, compared with those of the other kinds of devices, leads to much smaller current which is more susceptible to the inherent measurement error, resulting in fluctuations in the IV curves. Figure 5(d) shows the LR and HR for 20 consecutive sweeps. The cell shows stable switching endurance and the resistance ratio between the HRS and the LRS is larger than 10^8^. The much larger resistance ratio than that of the Ag/Ge_2_Sb_2_Te_5_/Pt cell, even under larger junction area, may be understood by the larger resistance of the GeS film as expected due to its wider band gap than that of Ge_2_Sb_2_Te_5_. The switching of Ag/GeS/Pt cell can also be understood by the typical ECM mechanism, namely, the field dependent redox reactions and ion movement.

**Figure 6:**
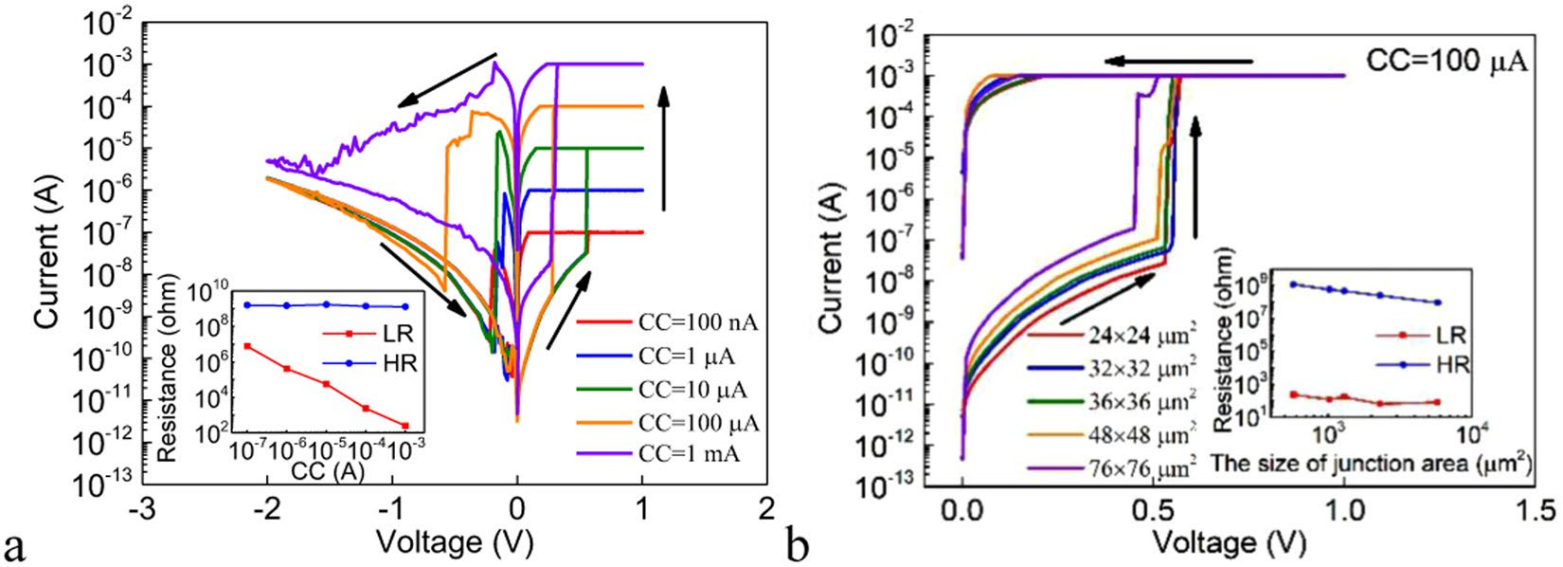
(**a**) The DC I-V curves under the different SET CCs for the Ag/GeS/Pt cell. Inset: the dependence of the LR and HR on the CC for the Ag/GeS/Pt cell. (**b**) The DC I-V curves in the SET process for Ag/GeS/Pt devices with different sizes of the junction areas under the SET CC of 100 μA. Inset: the dependence of the LR and HR on the size of the junction area for the Ag/GeS/Pt cell.

Figure 6(a) shows the DC I-V characteristics under different SET CCs. The RESET currents and the corresponding SET CCs are of the same order of magnitude, which is similar to the Ag/Ge_2_Sb_2_Te_5_/Pt cell. The inset shows that the LR decreases with increasing CC by forming stronger conductive filament(s) under higher CC. It is noted that when the CC is low (below 100 μA), the RESET shows abrupt behavior whereas when the CC is high (1 mA), the RESET shows gradual behavior. Therefore, we study the dependence of the HR on the NSV under both circumstances of CC = 100 μA (Fig. S1(e,f)) and CC = 1 mA (Fig. S1(g,h)). Under the former circumstance, the obtained HR shows no dependence on the NSV. This is expected from the abruptness of the RESET switching. Under the latter circumstance, the obtained HR of the device increases exponentially with increasing NSV, as the result of the gradual nature of the RESET switching. Figure 6(b) shows the DC I-V characteristics in the SET process for devices with different sizes of the junction areas under the SET CC of 100 μA. It is found that the cells switch to the LRS at ~ + 0.5 V regardless of the sizes of the junction areas. The inset of Fig. 6(b) shows the dependence of the LR and HR on the size of the junction area. The HR decreases with increasing size of the junction area, whereas the LR remains almost constant which can be attributed to the filamentary nature of the resistive switching.

**Figure 7:**
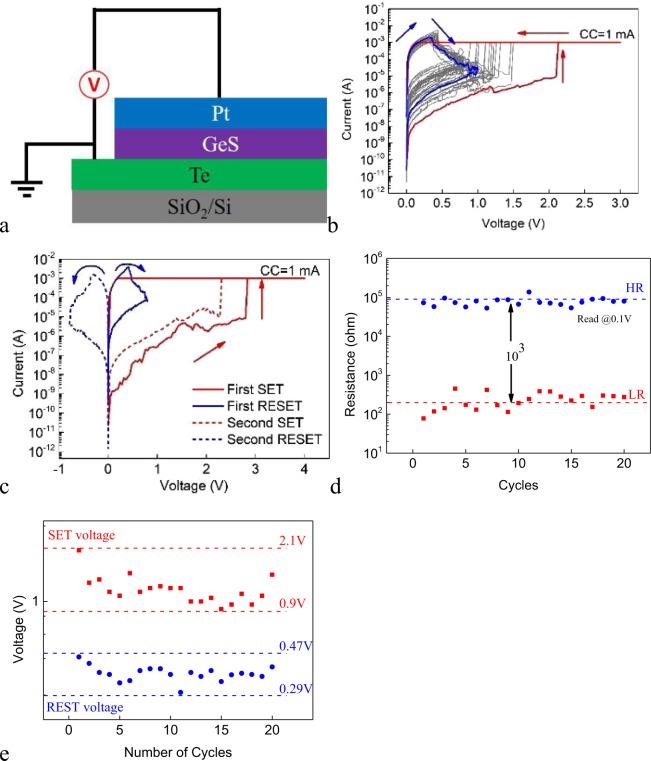
(**a**) The schematic structure of the Pt/GeS/Te cell. (**b**) The DC I-V curves of 20 consecutive sweep cycles for the Pt/GeS/Te cell under the SET CC of 1 mA. (**c**) The DC I-V curves of the sweeping only in one direction and in both directions for the Pt/GeS/Te cell. The SET CC is 1 mA. (**d**) The statistical distribution of the SET voltage and RESET voltage for the Pt/GeS/Te cell. (**e**) The LR and HR of the Pt/GeS/Te cell for 20 consecutive sweep cycles. The voltage of read pulse is 100 mV.

The Pt/GeS/Te cell is also fabricated to investigate the electrolyte dependent switching characteristics of ECM cell with a-AE. Figure 7(a) shows the schematic structure of the Pt/GeS/Te cell. Figure 7(b) shows the DC I-V curves of 20 consecutive sweep cycles under the SET CC of 1 mA for cell with 24 × 24 μm^2^ junction area. Similar to the Pt/Ge_2_Sb_2_Te_5_/Te cell, the SET process occurs under negative (positive) voltage polarity relative to the AE (CE), opposite to their Ag c-AE counterparts. The device requires electroforming. The cell switches to the LRS abruptly at certain voltage of larger absolute value than in the Ag/GeS/Pt cell. The AE dependence of the SET voltage is qualitatively similar to the Ge_2_Sb_2_Te_5_ electrolyte based cells. No resistive switching occurs if positive voltage on the a-AE is applied to the pristine device (not shown). Most strikingly different switching behavior than the other types of cells aforementioned is observed during the RESET process. Consecutive voltage sweep in the SET direction is found to be able to switch the cell back to the HRS in a gradual manner. The RESET can also occur by reversing the voltage polarity, as is shown in Fig. 7(c). The switching is repeatable. Figure 7(d) shows the LR and HR for 20 sweeps. It can be seen that the cell shows stable switching endurance and the resistance ratio between the HRS and the LRS is larger than 10^3^. Figure 7(e) depicts the distribution of the SET voltage and RESET voltage of the cell. The SET voltage varies in the range between 0.9 V–2.1 V while RESET voltage varies in the range between 0.29 V–0.47 V. These values of SET voltage and RESET voltage are non-overlapping, which is essential for memory applications. We interpret the anomalous unipolar switching of Pt/GeS/Te cell as the result of a SET process by ECM mechanism and a RESET process driven by Joule heating: when negative voltage is applied on the Te a-AE, Te is electrochemically reduced to Te^2−^ anions which migrate toward the Pt CE under the electric filed and are subsequently oxidized back to Te and nucleate, leading to the formation of Te semiconducting filament(s) and the decrease of cell resistance. During the RESET, the current through the conducting path is large enough that the generated Joule heat is sufficient to rupture the filament, irrespective of the voltage polarity. This can be viewed from Fig. 7(b) where the RESET current is about one order of magnitude larger than the SET CC. The large RESET current could be due to the formation of strong filament during the SET process. The switching mechanisms are schematically shown in Fig. S4.

**Figure 8:**
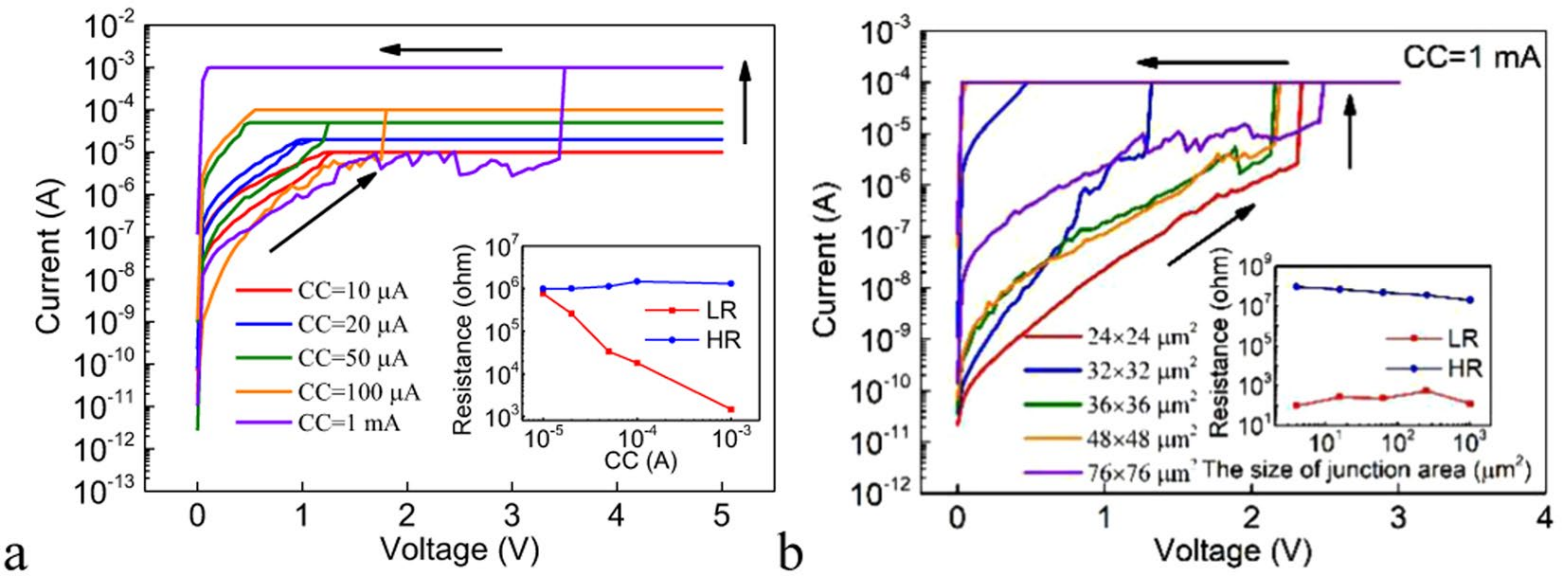
(**a**) The DC I-V curves under different SET CCs in the SET process for the Pt/GeS/Te cell. Inset: the dependence of the LR and HR on the CC. (**b**) The DC I-V curves under the SET CC of 1 mA in the SET process for Pt/GeS/Te cell devices with different sizes of the junction areas. Inset: the dependence of the LR and HR on the size of the junction area for the Pt/GeS/Te cell.

Figure 8(a) shows the DC I-V characteristics under different SET CCs and the inset shows the dependence of the LR and HR on the CC. It can be seen that the LR decreases with increasing CC, indicating the formation of stronger filament(s) under higher CC. The relationship between the HR and the PSV during RESET (R-PSV) is also investigated (Fig. S1(i,j)). Under small NSV, there is no RESET phenomenon and the device remains in the LRS. Under large enough NSV that allows the RESET to occur, the HR of the device increases exponentially with increasing NSV. This results from the gradual nature of the RESET switching, as can be seen from Fig. 7(b). Figure 8(b) shows the DC I-V characteristics in the SET process for devices with different sizes of the junction areas under the SET CC of 1 mA. It can be seen that the cells switch to the LRS at certain voltage in the range between 1.2 V and 2.5 V. The inset of Fig. 8(b) shows the dependence of the LR and HR on the size of the junction area. The LR is almost independent of the size of the junction area whereas the HR increases with decreasing the size of the junction area, which can be attributed to the filamentary nature of the resistive switching.

## Conclusions

In conclusion, electrochemical metallization cells with anion supplying active electrodes are fabricated and the electrolyte dependent switching characteristics are studied. The SET processes of both Pt/Ge_2_Sb_2_Te_5_/Te and Pt/GeS/Te cells occur under negative voltage on the Te electrodes, indicating that Te supplies anions by the ECM mechanism. The electrical properties, such as the SET voltages, for both cells are different from those of the corresponding device counterparts with Ag active electrodes, which is expected from the unique chemical natures of semiconducting Te. Moreover, we observe different modes of switching between the two types of cells, namely, unipolar switching for Pt/GeS/Te cell and bipolar switching for Pt/Ge_2_Sb_2_Te_5_/Te cell. The different switching modes can be attributed to the rupture of the Te filament during RESET by Joule heating for Pt/GeS/Te cell and by ECM for Pt/Ge_2_Sb_2_Te_5_/Te cell, respectively. This work provides a springboard for more studies on this new modality of the ECM memory cells and opens up unprecedented applications based on the integrated multi-functionalities of Te.

## Method

Si (ASM, Netherlands) with 300 nm thermally grown SiO_2_ is used as the substrate for the thin film deposition. Prior to the deposition of the thin films, the substrate is cleaned in acetone under ultrasonic agitation, then rinsed in isopropanol, and dried under nitrogen flow. All the films are deposited using a magnetic sputtering system (AJA, USA) at room temperature in 20 sccm (standard cubic centimeters per minute) Argon (Ar) atmosphere.

For the Ag(20 nm)/Ge_2_Sb_2_Te_5_(40 nm)/Pt(20 nm) cell, 5 nm Ti adhesion layer and Pt bottom electrode are fabricated by photolithographic patterning followed by magnetron sputtering and lift-off. Then Ge_2_Sb_2_Te_5_ electrolyte layer, Ag top electrode and 100 nm Pt antioxidation layer are fabricated to form the cross-point electrode structure, using the same procedure. Different junction areas (2 × 2, 4 × 4, 8 × 8, 16 × 16 and 32 × 32 μm^2^) are obtained. Similarly, Pt(100 nm)/Ge_2_Sb_2_Te_5_(40 nm)/Te(20 nm) cells are fabricated. On the other hand, Pt(100 nm)/GeS(4;0 nm)/Pt(20 nm), Ag(20 nm)/GeS(40 nm)/Pt(20 nm) and Pt(100 nm)/GeS(40 nm)/Te(20 nm) cells are fabricated by only one photolithographic patterning to obtain different junction areas (24 × 24, 32 × 32, 36 × 36, 48 × 48 and 76 × 76 μm^2^).

Electrical characterization of the devices is performed using Keysight B1500A semiconductor cell analyser, which is equipped with high-resolution source and measurement units to sense the current with an integration time of 16 power line cycles with a specified resolution of 1 fA. All electrical measurements are performed at room temperature and under an ambient atmosphere.

## Electronic supplementary material

Supplementary Information
