# Supplementary material for: Electrochemical metallization cell with anion supplying active electrode
Source: Sci Rep. 2018 Aug 22;8:12617. doi: 10.1038/s41598-018-30746-6 (PMC6105648; doi:10.1038/s41598-018-30746-6)
Supplement: Supplementary file 1 — Supplementary Information [file 41598_2018_30746_MOESM1_ESM.docx]

*Supplementary Information*

**Electrochemical metallization cell with anion supplying active electrode**

*Ziyang Zhang^1+^, Yaoyuan Wang^1+^, Yan Luo^2+^, Yuhan He^2^, Mingyuan Ma^2^, Rongrong Yang^1^, Huanglong Li^1^**

^1^ Department of Precision Instrument, Center for Brain Inspired Computing Research, Tsinghua University, China, ^2^ Department of Electronic Engineering, Tsinghua University, China

**Corresponding author**

*Email: [li_huanglong@mail.tsinghua.edu.cn](mailto:li_huanglong@mail.tsinghua.edu.cn)

a
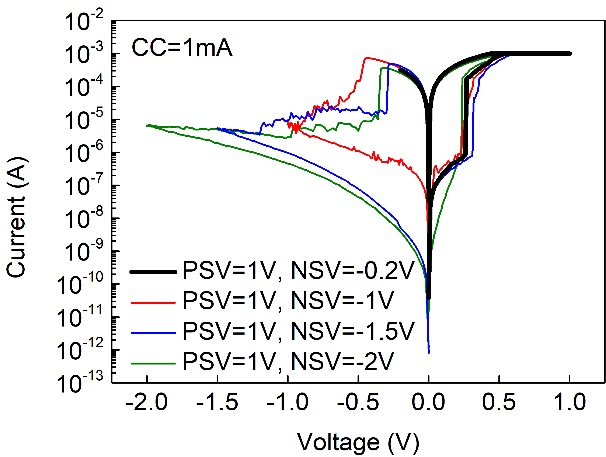
b
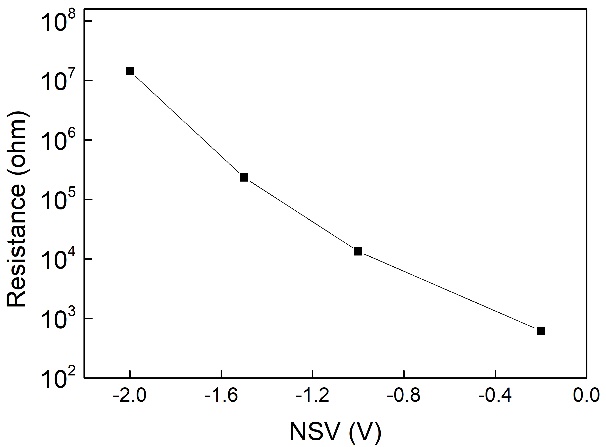


c
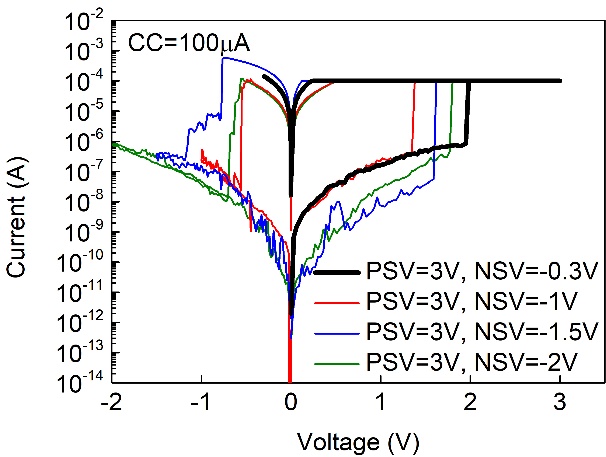
d
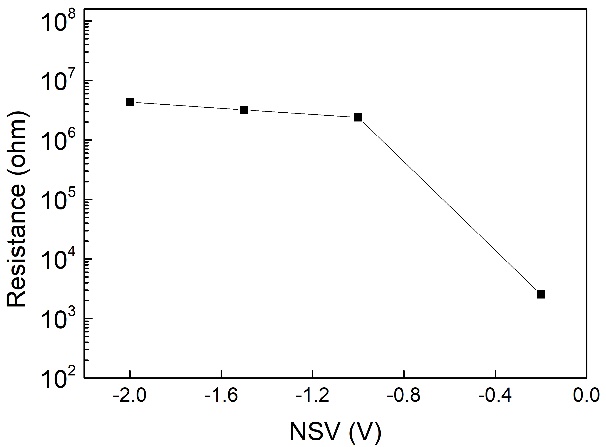


e
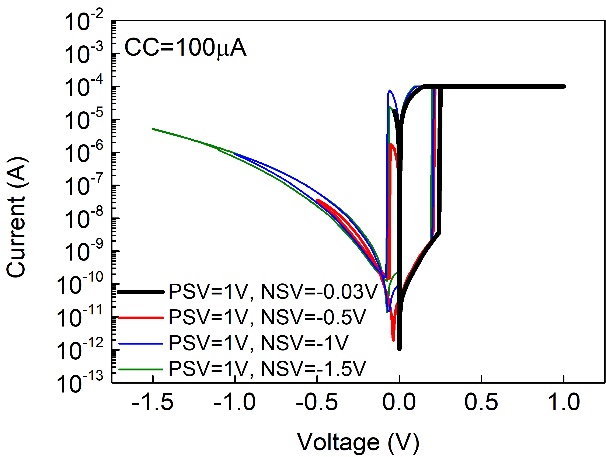
f
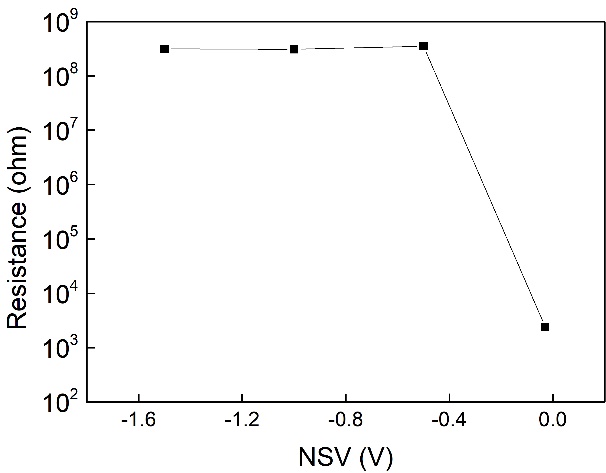


g
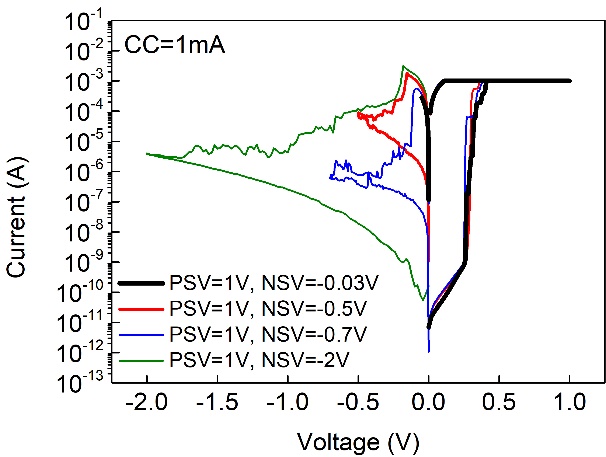
h
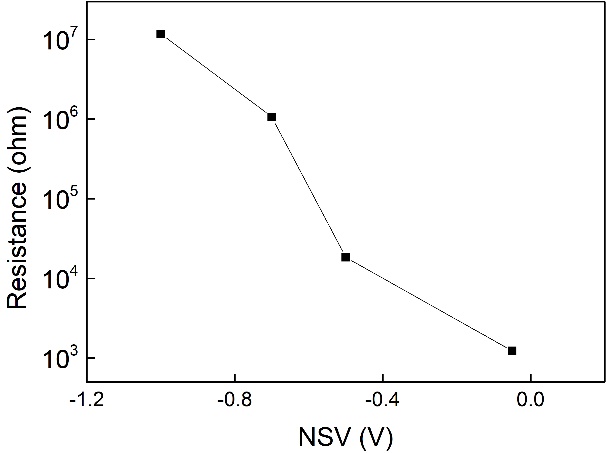


i
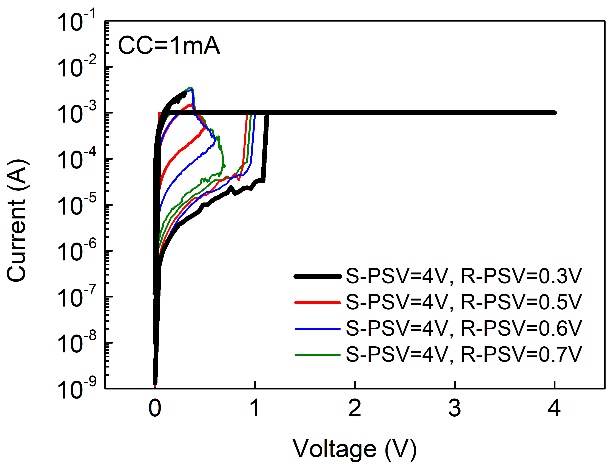
j
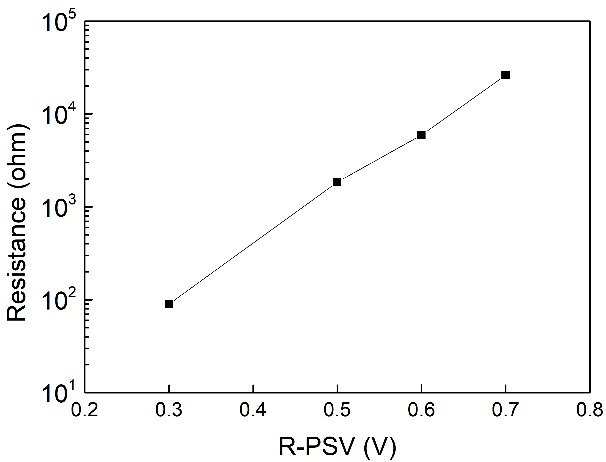


**Figure S1.** a) The DC I-V curves for different NSVs applied on the Ag/GST/Pt cell. The PSVs are fixed to 1 V and the CCs are fixed to 1 mA. The positive direction in the diagrams of this work corresponds to positive voltage on the top electrode. b) The relationship between the resistance of the Ag/ GST/Pt device and the NSV. c) The DC I-V curves for different NSVs applied on the Pt/GST/Te cell. The PSVs are fixed to 3 V and the CCs are fixed to 100 μA. d) The relationship between the resistance of the Pt/GST/Te device and the NSV. e) The DC I-V curves for different NSVs applied on the Ag/GeS/Pt cell. The PSVs are fixed to 1 V and the CCs are fixed to 100 μA. f) The relationship between the resistance of the Ag/GeS/Pt device and the NSV under the circumstance of CC=100 μA. g) The DC I-V curves for different NSVs applied on the Ag/GeS/Pt cell. The PSVs are fixed to 1 V and the CCs are fixed to 1 mA. h) The relationship between the resistance of the Ag/GeS/Pt device and the NSV under the circumstance of CC=1 mA. i) The DC I-V curves for different R-PSVs applied on the Pt/GeS/Te cell. The S-PSVs are fixed to 4 V and the CCs are fixed to 1 mA. j) The relationship between the resistance of the Pt/GST/Te device and the R-PSV.


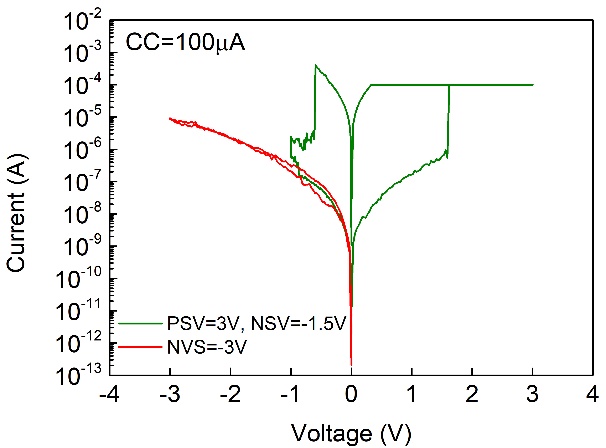


**Figure S2.** The DC I-V curves under different voltage sweepings for the Pt/GST/Te cell. Red: the voltage sweep starts in the negative direction. Green: the voltage sweep starts in the positive direction.

Figure S2 shows the switching behaviors of the pristine Pt/GST/Pt cell under different voltage sweepings. It can be seen that no switching occurs if the voltage sweep starts in the negative direction (negative voltage on the top Pt electrode), even if the NSV is as large as -3 V. This is an indication that the GST electrolyte itself cannot be responsible for the observed switching in our devices. However, when the voltage sweep starts in the positive direction (positive voltage on the top Pt electrode), SET switching occurs. In this case, the device can also be reversibly reset back to the HRS by reversing the voltage polarity applied on the device. It is noteworthy that the switching characteristics are quite similar to those observed for the Pt/GST/Te device, as shown in figure 3b of the manuscript, including the values of the SET and RESET voltages. Yo et al [1] has reported the resistive switching behavior of the Pt/GST/Pt device and the switching has been attributed to the unintentional formation of Te layer at the interface of GST and the bottom Pt electrode during the fabrication. Our Pt/GST/Pt device shows the same switching polarity as Yo’s. Therefore, it is reasonable to attribute the observed switching in our Pt/GST/Pt device to the formation of Te anion supplying layer.

According to the above analyses, it is possible that an additional Te layer is formed at the interface of GST and Pt in our Ag/GST/Pt device, serving as an anion supply counter electrode. By comparing with the Pt/GST/Te and Pt/GST/Pt devices, however, we believe that the switching of the Ag/GST/Pt device is not related to the Te layer, if any. Pt/GST/Te and Pt/GST/Pt devices require much higher voltage (~ 2 V) to switch on, whereas Ag/GST/Pt device can be set by voltage less than 0.5 V at which the electrochemical growth of the Te filament is not expected to take place. In other words, Te counter electrode becomes inert in the presence of Ag electrode.

a
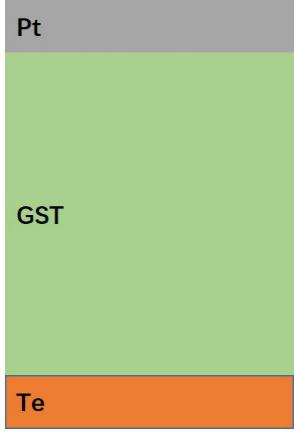
b
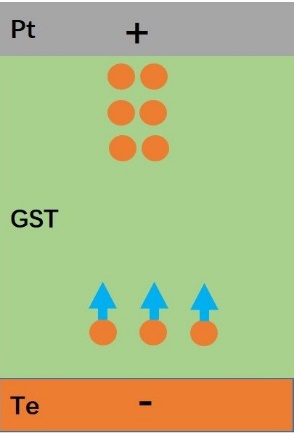
c
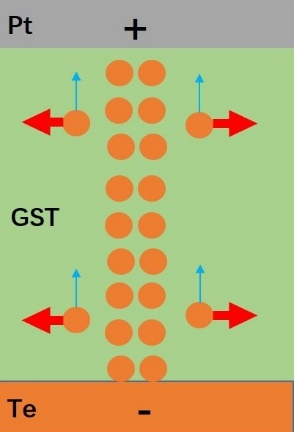
d
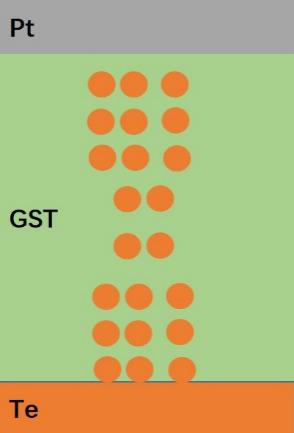


e
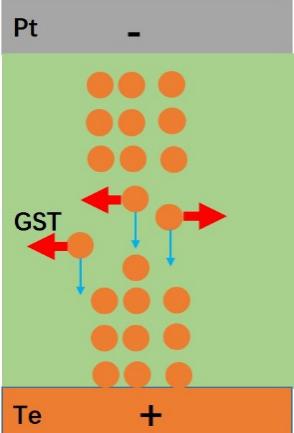
f
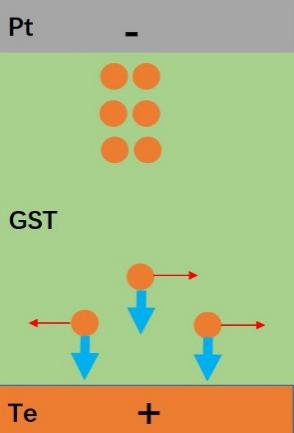
g
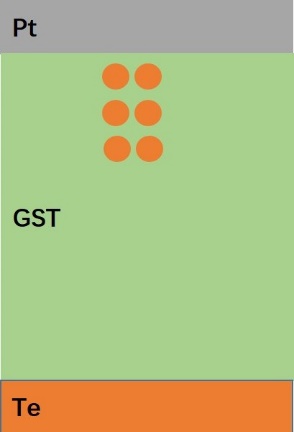


**Figure S3**. The switching mechanisms proposed for the Pt/GST/Te cell. (a-d) are for the SET process and (c-g) are for the RESET process.

Figure S3(a) shows that the pristine Pt/Ge_2_Sb_2_Te_5_/Te device is insulating without pre-existed conducting path. When a negative bias is applied to the Te active electrode (AE). The AE is electrochemically reduced and releases cations which then drift across the electrolyte toward the Pt inert electrode (IE) under the electric field, and are subsequently electrochemically reduced and nucleate, leading to the formation of (semi)conducting filament within the electrolyte, as is shown in figure S3(b). The formation of the conducting path results in abrupt increase of the current and local temperature increase due to the joule heating effect. While the drift or electro-migration under the electric field results in vertical motion of ions along the filament, the temperature gradient contributes to lateral atomic diffusion which results in the formation of stronger filament, as is shown in figure S3(c)-(d). If then a large enough voltage of opposite polarity is applied, a reversed electrochemical process occurs to dissolve the filament. At the same time, due to the existence of an initial conducting path, the local temperature increases due to the Joule heating effect which facilitates the disconnection of the filament by the diffusion of Te into the surroundings under their concentration gradient, as is shown in figure S3(e)-(g).

a
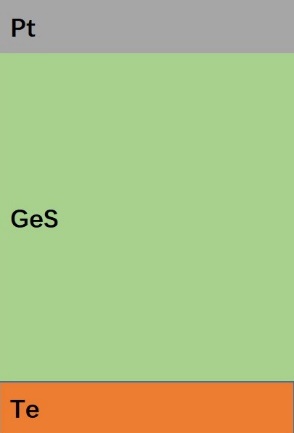
b
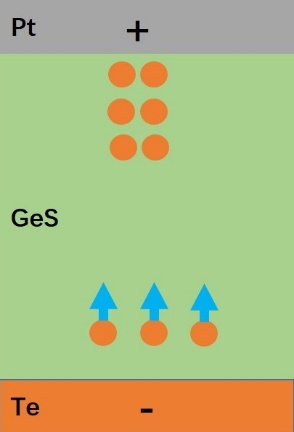
c
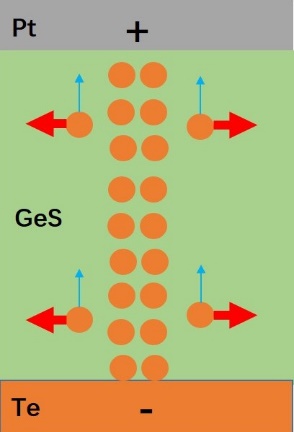
d
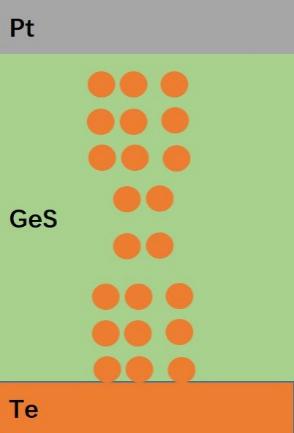


e
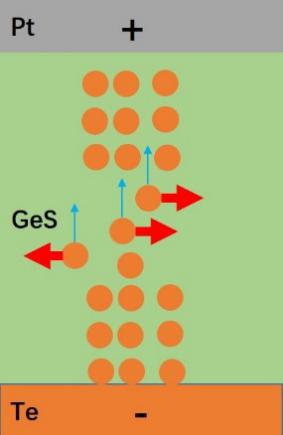
f
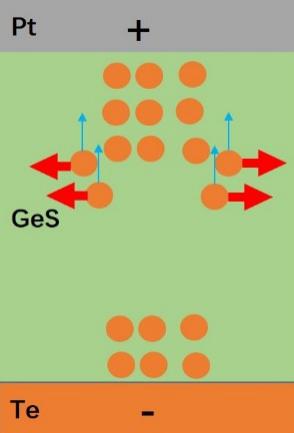
g
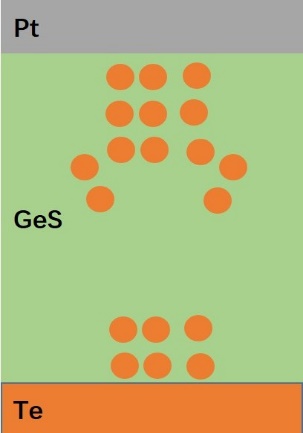


**Figure S4.** The switching mechanisms proposed for the Pt/GeS/Te cell. (a-d) are for the SET process and (c-g) are for the RESET process.

Figure S4 shows the proposed switching mechanisms for the Pt/GeS/Te cell. The main difference can be seen in the RESET process. Irrespective of the voltage polarity, the current through the conducting path is large enough that the generated Joule heat is sufficient to rupture the filament at its relatively weak point. The large RESET current could be due to the formation of strong filament during the SET process. As the electrochemical dissolution of the Te filament will require voltage polarity in the opposite direction, the observed RESET process under the same polarity as that for the SET process indicates that the RESET can be driven by the Joule heating effect alone.

**References**

[1] Yo, S., Eom, T., Gwon, T. & Hwang, C. S. Bipolar resistive switching behavior of an amorphous Ge_2_Sb_2_Te_5_ thin films with a Te layer. *Nanoscale* **7**, 6340-6347 (2015).
